# Reversed Expression of the JAK/STAT Pathway Related Proteins Prolactin Receptor and STAT5a in Normal and Abnormal Breast Epithelial Cells

**DOI:** 10.4137/bcbcr.s549

**Published:** 2008-02-26

**Authors:** Gary L. Bratthauer, Brian L. Strauss, Ross Barner

**Affiliations:** 1 Department of Gynecologic and Breast Pathology, Armed Forces Institute of Pathology, Washington; 2 Quest Diagnostics Incorporated, Las Vegas NV; 3 Department of Pathology, Walter Reed Army Medical Center, Washington DC

**Keywords:** STAT5a, prolactin receptor, immunohistochemistry, breast, secretory

## Abstract

The JAK/STAT pathway is important for cellular metabolism. One component, STAT5a, is activated in the breast upon prolactin to prolactin receptor (PRLR) binding facilitating the transcription of genes involved in lobule development. STAT5a was previously found to be expressed in most normal breast epithelial cells but not in many *in situ* or invasive carcinomas except secretory carcinomas which retain STAT5a expression. This report examines the JAK/STAT pathway in the breast through the detection of PRLR and STAT5a. Fifty breast tissues, including benign secretory change, microglandular adenosis, usual and atypical hyperplasia and *in situ* and invasive ductal carcinoma both usual and secretory, were obtained from the files of the Armed Forces Institute of Pathology. Sections were immunostained with antibodies to PRLR and STAT5a. PRLR was minimally detected on the surface of a few normal breast epithelial cells whereas STAT5a was greatly expressed in over 80% of normal cell nuclei. PRLR was also minimally detected in secretory carcinomas expressing STAT5a. However, the opposite pattern was seen in breast carcinomas lacking STAT5a expression. PRLR was abundantly expressed in these cells. This reversed expression may indicate a JAK/STAT pathway disturbance that could play a role in the initiation or maintenance of an abnormal breast phenotype.

## Introduction

The seven signal transducer and activator of transcription (STAT) molecules are effectors of hormonal and cytokine stimulation through the transcription of a variety of regulatory and differentiation proteins. The STATs are activated through the signaling of receptor-ligand complexes. One of the STAT proteins, STAT5a, is found in many cell types including breast epithelial cells where it is activated by the binding of prolactin to the prolactin receptor (PRLR). Activated STAT5a then undergoes dimerization, nuclear transport, and DNA binding, facilitating the transcription of genes involved in lobule development and mammary gland differentiation (Clevenger, 2004). STAT5a-deficient transgenic mice develop normally, except for defective mammary gland development (Akira, 1999) and signaling via the PRLR through STAT5a is essential for lobuloalveolar growth and differentiation during pregnancy (Myoshi et al. 2001).

Using a monoclonal antibody, we have shown in formalin-fixed, paraffin-embedded, breast tissue ranging from simple hyperplasia to invasive carcinoma that a direct correlation exists between abnormalities in breast epithelial cells and reduction in STAT5a expression, with STAT5a expression being decreased in ductal carcinoma *in situ* (DCIS) and invasive ductal carcinoma (Bratthauer et al. 2006). The reduction of STAT5a may result in aberrant signaling of developmental or regulatory proteins, contributing to altered (metaplastic or neoplastic) differentiation. Cells undergoing secretory change over-express STAT5a. Surprisingly, secretory carcinomas of the breast maintain STAT5a expression, differing from other potential mimics such as apocrine metaplasia and mucinous or clear cell carcinomas, none of which expressed STAT5a in this study (Strauss et al. 2006).

Whether STAT5a affects the maintenance of the normal breast phenotype, as it does the establishment of that phenotype, is subject to conjecture. The Janus kinase (JAK)/STAT pathway is important to cell development and differentiation; defects can lead to inhibition of growth restraint, with prolactin implicated in the pathogenesis of human breast cancer (Clevenger et al. 2003). Because the JAK/STAT pathway is integral to the function of normal breast epithelial cells, abnormalities in this pathway between prolactin and STAT5a could play a role in the initiation or maintenance of carcinoma. This report examines this pathway by comparing PRLR and STAT5a expression patterns in benign and malignant examples of secretory and non-secretory breast epithelium.

## Materials and Methods

Fifty formalin-fixed, paraffin-embedded, tissues representing varying breast disease were obtained from the files of the Armed Forces Institute of Pathology (AFIP). All material was part of research protocols approved by the AFIP’s Institutional Review Board. The samples included usual and atypical ductal hyperplasia, microglandular adenosis, *in situ* and invasive ductal carcinoma, secretory carcinoma, and those exhibiting secretory/lactational change. Most of the samples also contained normal terminal duct lobular units (TDLU). Tissues were selected based on STAT5a results obtained previously (Bratthauer et al. 2006). Tissues expressing and not expressing the STAT5a protein were assayed with antibody-optimized immunohistochemical protocols (Bratthauer, 1999) using antibodies to PRLR and STAT5a. Briefly, 6 micron sections were heated for 30 minutes at 70 °C and then deparaffinized along with antigen enhancement in a solution of Reveal (Biocare Medical Corporation, Concord, CA) in a pressure cooker for 3 minutes at 20 psi added pressure. Endogenous oxidative compounds were quenched with a 30 minute incubation in 10% H_2_O_2_ in methanol. Monoclonal antibody ST5a-2H2 reactive against STAT5a (Zymed Laboratories, South San Francisco, CA), and monoclonal antibody B6.2, reactive against PRLR (Neomarkers, Lab Vision Corporation, Fremont CA), diluted 1:400 and 1:100 respectively in TBS Plus (Biocare) with 0.1% Tween 20 (DakoCytomation, Carpinteria, CA) (TBST), were applied to the sections for 60 minutes at room temperature. Concentrations were determined by serial dilution on known positive specimens. Detection was with the Elite Avidin-Biotin Complex (ABC) system (Vector Laboratories, Burlingame CA) using biotinylated horse anti-mouse/rabbit IgG followed by ABC reagent applied for 45 minutes each with intervening rinses of TBST. Development occurred for 12 minutes using 0.08% diaminobenzidine tetrahydrochloride (Sigma Chemical Co., St. Louis, MO) with 0.024 H_2_O_2_ (Sigma). Sections were counterstained with hematoxylin, dehydrated in ethanol, and mounted in Permount (Fisher Scientific Corporation, Pittsburgh, PA) following immersion in xylene. Simultaneous identification of PRLR and STAT5a was performed by double-labeling immunohistochemistry as described earlier (Bratthauer et al. 2002). Diluted normal sera in substitution for the primary antibody served as a negative control. Selected positive and negative tissues were analyzed with alternate antibodies, a polyclonal rabbit antiserum directed against STAT5a (L-20, Santa Cruz Biotechnology Inc, Santa Cruz, CA, diluted 1:100) and anti-PRLR clone SPM213 (Abcam Inc., Cambridge, MA, undiluted).

To assess the specificity for STAT5a, a STAT5a peptide (Neomarkers, LabVision) was added to antibody ST5a-2H2 and allowed to incubate prior to antibody application. Graded concentrations of protein (0.1, 1.0 and 10 ug/ml) were added to antibody solutions which were incubated at 37 °C overnight, then centrifuged at 6000 rpm for 5 minutes and applied to STAT5a positive control tissue sections. A non-related protein was used in parallel absorption experiments as a control.

To assess the efficacy of the PRLR detection, enzyme enhanced antigen pretreatments were compared to the above method of heat retrieval in a pressure cooker. Both pepsin (Sigma), at 1.0 mg/ml for 10 minutes at 37 °C in a Tris-HCl pH 2 solution, and protease VIII (Sigma), at 0.01 g/ml in TBST at 37 °C for 5 minutes, were used as alternate pretreatments following tissue rehydration.

Assayed sections were examined for extent of immunostaining in a double-blind fashion by two of us (GLB, BLS). Tissues were scored as a product of intensity and distribution. Intensity was scored as 1+ (weak), 2+ (moderate), or 3+ (strong). The intensity score was multiplied by a distribution score of 1+ (5%–10%), 2+ (11%– 40%), 3+ (41%–75%), or 4+ (76%–100%), to arrive at a semi-quantitative immunoscore ranging from 0 to 12.

## Results

**Figure 1.**
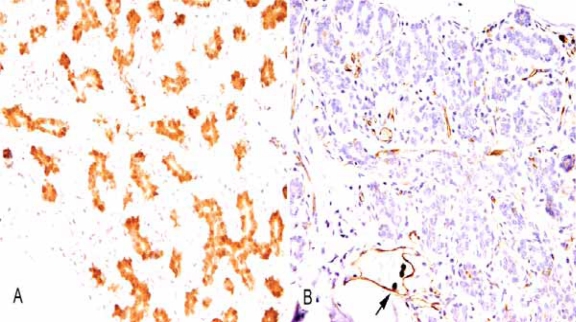
**A**) Immunohistochemical demonstration of STAT5a in the cytoplasm and nucleus of normal breast TDLU (200X). **B**) Immunohistochemical demonstration of PRLR in this TDLU (200X). PRLR reactivity is mainly associated with endothelial cells and neutrophils (arrow).

The results are listed in Table 1. The expression of STAT5a corresponded to earlier studies (Bratthauer et al. 2006) with the majority of normal lobular and ductal epithelial cells demonstrating STAT5a in the cytoplasm and nucleus (Fig. 1A). The cells of usual ductal hyperplasia and microglandular adenosis also expressed STAT5a and internal controls of adipose tissue, endothelial cells, and lymphocytes always showed reactivity. STAT5a expression was occasionally reduced in larger ducts and cells undergoing apocrine differentiation did not show any STAT5a expression.

In contrast, normal epithelium, along with usual ductal hyperplasia and microglandular adenosis, generally showed only minimal detectable PRLR (Fig. 1B), present as the luminal surface labeling of occasional cells. Neutrophils, endothelial cells, and smooth muscle cells (which normally show cytoplasmic PRLR) (Camarillo et al. 2001; Dogusan et al. 2001; Merkle et al. 2000; Nowak et al. 1999) were reactive and served as internal controls. Cells undergoing apocrine differentiation demonstrated cytoplasmic PRLR reactivity as did scattered myoepithelial cells in larger ducts.

**Figure 2.**
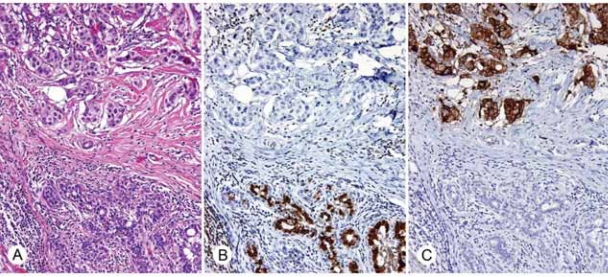
**A**) H&E stain of invasive ductal carcinoma of the breast (top) adjacent to normal duct (bottom) (200X). **B**) Immunohistochemical demonstration of STAT5a expression (200X). **C**) Immunohistochemical demonstration of PRLR expression (200X). STAT5a is expressed in the normal duct and absent in the invasive ductal carcinoma; in contrast, PRLR is expressed in the invasive ductal carcinoma and absent in this normal duct.

**Figure 3.**
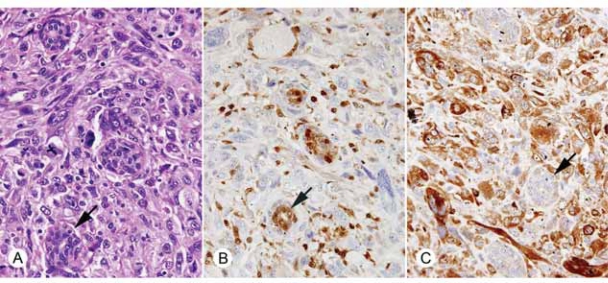
**A**) H&E stain of invasive ductal carcinoma of the breast (400X) with occasional entrapped normal ducts (arrow). **B**) Immunohistochemical demonstration of STAT5a in normal ducts (arrow) and scattered endothelial cells and lymphocytes (400X). STAT5a is absent in the tumor cells. **C**) Immunohistochemical demonstration of PRLR in the invasive ductal carcinoma (400X). Entrapped normal ducts show reduced PRLR expression (arrow).

As was shown previously, abnormal breast epithelium does not generally express the STAT5a protein using antibody ST5a-2H2 (Bratthauer et al. 2006). For this study, specimens previously shown to have ADH, DCIS, or invasive carcinoma only minimally expressing STAT5a (mean immunoscores 1.0–1.2) were examined for PRLR. Epithelial cells that essentially lacked STAT5a showed abundant PRLR expression in the cytoplasm as well as the cell membrane (Figs. 2 and 3), with mean immunoscores ranging from 10.4 to 11.0.

**Figure 4.**
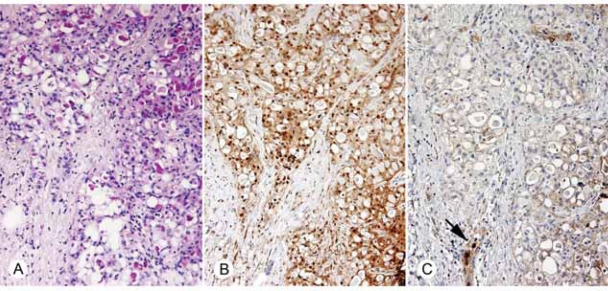
**A**) H&E stain of secretory carcinoma of the breast (400X). **B**) Immunohistochemical demonstration of retained STAT5a (400X). **C**) Immunohistochemical detection of diminished PRLR expression in this secretory carcinoma (400X). Arrow denotes neutrophil control.

Secretory carcinomas tend to retain STAT5a expression (Strauss et al. 2006), and specimens with either benign secretory change or secretory carcinoma (mean STAT5a immunoscores of 11.4 and 9.2, respectively) demonstrated greatly reduced PRLR (mean immunoscores of 1.2 and 2.7, respectively) (Fig. 4).

**Figure 5.**
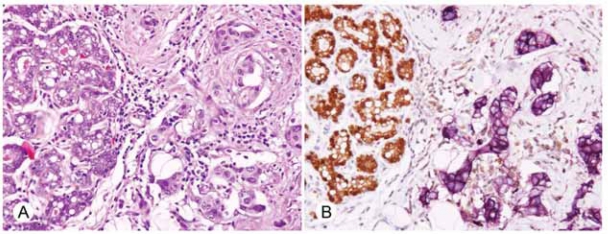
**A**) H&E stain of invasive ductal carcinoma, NOS (400X), showing normal cells with secretory change (left) adjacent to invasive tumor (right). **B**) Simultaneous immunohistochemical demonstration of STAT5a (brown) and PRLR (purple) as they are expressed in these normal and abnormal cells (400X).

In almost all the cases examined, both normal and abnormal epithelia expressed either STAT5a or PRLR, with only a few cells expressing either both proteins or neither protein. This was highlighted by two-color simultaneous labeling (Fig. 5). All negative controls were non-reactive.

Assays with alternate antibodies to STAT5a and PRLR (L20 and SPM213, respectively) essentially demonstrated these same patterns of reactivity (data not shown) with antibody L20 exhibiting slightly increased background staining.

In addition, alternate methodology for detecting PRLR employing either protease or pepsin digestion showed the same reactivity patterns as the heat and pressure antigen recovery method. Preincubation with peptide demonstrated that STAT5a peptide added at 0.1ug/ml to ST5a-2H2 effectively extinguished all STAT5a observed reactivity, while a non-related protein added at up to 10 ug/ml had no effect (data not shown).

## Discussion

Prolactin regulates mammary gland development during organogenesis, pregnancy and lactation (Horseman, 1999). In the breast it activates STAT5a, necessary for normal lobular growth. We have previously shown STAT5a expression reduced in many abnormal and malignant breast duct epithelial cells (Bratthauer et al. 2006) and were interested in further investigating the prolactin-initiated JAK/STAT pathway and its relationship to abnormal breast epithelial cell growth.

In examining normal and hyperplastic breast epithelium, we noted that where STAT5a was present immunohistochemically, PRLR expression was much diminished. Conversely, in most abnormal or malignant breast epithelial cells, wherever STAT5a was absent, PRLR was markedly increased. A consistent exception was found in secretory carcinomas of the breast; these paralleled normal and hyperplastic breast epithelial cells in STAT5a and PRLR expression.

Antibody ST5a-2H2, recognizing STAT5a, reacts with the nuclei of most normal breast epithelial cells, and only rarely so with abnormal epithelium (Bratthauer et al. 2006). As outlined previously, published accounts vary in the degree of STAT5a positivity reported in human breast cancer, with one study indicating a 17% positivity rate for STAT5a in breast cancers (Nevalainen et al. 2004) (approximating the rate found in our article), and another demonstrating STAT5a in 76% (Cotarla et al. 2004). In this current paper, the specificity of the STAT5a immunostaining profile is supported by demonstration of essentially the same pattern with an alternate antibody (L20), as well as the blocking of all reactivity with a STAT5a peptide.

Antibody B6.2 has been shown to identify PRLR in formalin-fixed, paraffin-embedded, breast tissues (Gill et al. 2001). Using this and other antibodies, researchers have identified PRLR expression to some degree in most human breast cancers and normal breast tissue, on the luminal surface (Gill et al. 2001; Reynolds et al. 1997; Mertani et al. 1998). In the present study, although some luminal staining was observed in normal breast epithelium, a much stronger reactivity in DCIS and invasive carcinoma was observed throughout the cytoplasm and sometimes distinctly on the entire cell surface. The pattern was also observed with a different antibody (SPM213) using multiple different antigen retrieval methods, supporting the specificity of the PRLR immunoprofile obtained here. That this pattern differs from some earlier reports is likely due in part to differences between antibody specificities. Antibody U6 recognizes the extracellular domain of PRLR (Reynolds et al. 1997) while in another study a polyclonal goat antiserum showed partially perinuclear staining in normal and pregnant rats (Camarillo et al. 2001). Variable antigen specificity may be the cause as well since PRLR exists in several functional isoforms (Clevenger et al. 2003; Reynolds et al. 1997; Trott et al. 2003). Alternate patterns of reactivity can also stem from what was interpreted as positive immunostaining; in this report we have made a distinction between the often faint luminal expression of PRLR seen in normal breast tissue of the type previously reported (Gill et al. 2001) and the much stronger cytoplasmic immunostaining observed in most human breast carcinomas where STAT5a was not expressed.

The prolactin-JAK/STAT pathway is necessary for normal breast development and its possible involvement in breast oncogenesis is intriguing. The changes seen in this study in the reciprocal STAT5a/PRLR relationship with breast carcinoma may be due to signaling defects in this pathway. In normal cells, the nuclear location of STAT5a may provide evidence of its activation, as activation generally causes STAT5a to accumulate in the nucleus (Iyer and Reich, 2007). Combined with the over-expression of PRLR, lack of detectable STAT5a in the nuclei of *in situ* and invasive ductal carcinoma suggests the possibility of attempted STAT5a activation that has been inhibited. Abnormalities that could result in abundant PRLR and undetectable STAT5a include STAT5a mutations that produce a truncated, non-functional version (leading to constitutive PRLR expression) (Kazansky et al. 1995; Mui et al. 1995) and PRLR mutations incapable of activating STAT5a (resulting in over-accumulation of PRLR, as is the case with p53 mutations). While PRLR gene mutations have yet to be identified in the majority of breast cancers (Glasow et al. 2001), they exist in some patients (Canbay et al. 2004) and PRLR expression has been implicated in breast oncogenesis (Clevenger et al. 2003; Gill et al. 2001). Since PRLR exists in several functional isoforms (Clevenger et al. 2003; Reynolds et al. 1997; Trott et al. 2003) and the breast’s endogenous prolactin can amplify hormonal effects by up-regulating specific PRLR isoforms (Clevenger et al. 2003; Gutzman et al. 2004), these isoforms may interact differently with STAT5a. Recently, the demonstration of impaired PRLR degradation in breast tumors correlated with enhanced PRLR expression (Li et al. 2006).

Different forms of prolactin also have different effects on downstream signaling (Wu et al. 2003), and prolactin regulates a variety of transcription factors and other proteins affecting proliferation, such as AP-1, insulin like growth factor 2 and pim-1 (Borg et al. 1999; Brisken et al. 2002; Gutzman et al. 2005).

Other proteins related to PRLR-STAT signaling also may be involved. Post-transcriptional mechanisms down-regulate STAT5a through both the protein inhibitors of activated STATs (PIAS) and the suppressors of cytokine signaling (SOCS) proteins (Kotaja et al. 2000; Martens et al 2005). The tyrosine phosphatase SHP-2 has been shown to regulate the interaction of JAK2 and SOCS1 (Ali et al. 2003), and JAK2 itself, along with C/EBPbeta, can interfere with prolactin/STAT5 signaling (Grimm et al. 2002; Wagner et al. 2004). Also, while PRLR does not require an intact cytoskeleton for initial phosphorylation, cytoskeletal integrity is necessary to transduce signals from PRLR to STAT5a (Zoubiane et al. 2004). Recently it was shown that STAT5a could be epigenetically silenced by the tyrosine kinase NPM1-ALK resulting in the lack of reciprocal inhibition of this oncogenic kinase by STAT5a providing some evidence of a possible tumor suppression function for STAT5a (Zhang et al. 2007).

Intriguingly, secretory carcinomas of the breast retain the STAT5a expression seen in normal breast epithelium. As described previously, this could be due to retention of transcription factors that are lost during the development of other types of breast cancers, or related to a specific translocation others have described in secretory carcinomas, t(12; 15) (Tognan et al. 2002), that results in the fusion protein ETV6-Ntrk3 possibly activating STAT5a. Regardless of the reason for continued STAT5a expression, secretory carcinomas are similar to other STAT5a positive tissues in showing reduced PRLR expression. It may therefore be useful to compare the involvement of the prolactin-JAK/STAT pathway in secretory and non-secretory types of breast cancer.

In summary, STAT5a and PRLR show a reciprocal pattern of expression between normal and abnormal breast tissues. Reversal of the expression seen in normal cells in DCIS and invasive carcinoma, resulting in increased PRLR and decreased STAT5a, may indicate a signaling pathway disturbance and possibly a divergence related to carcinogenesis. Further investigation will be required to understand the mechanisms by which this may occur.

## Figures and Tables

**Table 1.** Relationship between STAT5a expression and PRLR expression in various breast epithelial cells.

**Type**	**# Total**	**STAT5a**		**PRLR**	

		**# Positive**	**Mean IS**	**# Positive**	**Mean IS**
Usual Ductal Hyperplasia	3	3	10.7	3	3.0
Microglandular Adenosis	1	1	4	0	0
Atypical Ductal Hyperplasia	2	1	1.0	2	10.5
Ductal Carcinoma in Situ	9	4	1.2	9	11.0
Invasive Ductal Carcinoma	20	8	1.0	20	10.4
Benign Secretory Changes	7	7	11.4	4	1.2
Secretory Breast Carcinoma	8	8	9.2	3	2.7

Immunoscore (IS) = intensity score × distribution score (range of 0–12).
